# In Vitro Modulation of Human Foam Cell Formation and Adhesion Molecules Expression by Ginger Extracts Points to Potential Cardiovascular Preventive Agents

**DOI:** 10.3390/ijms25179487

**Published:** 2024-08-31

**Authors:** Alessandro Scalia, Maxime Coquay, Nadège Kindt, Pierre Duez, Rania Aro, Fabrice Journé, Mathilde Fabjanczyk, Anne Trelcat, Stéphane Carlier

**Affiliations:** 1 Department of Cardiology, UMONS Research Institute for Health Sciences and Technology, University of Mons (UMONS), 7000 Mons, Belgium; alessandro.scalia@umons.ac.be (A.S.); coquay.maxime@alumni.umons.ac.be (M.C.); nadege.kindt@hubruxelles.be (N.K.); anne.trelcat@umons.ac.be (A.T.); 2 Department of Therapeutic Chemistry and Pharmacognosy, University of Mons (UMONS), 7000 Mons, Belgium; pierre.duez@umons.ac.be (P.D.); rania.aro@umons.ac.be (R.A.); mathilde.fabjanckz@alumni.umons.ac.be (M.F.); 3 Department of Clinical and Experimental Oncology, Institute Jules Bordet, Université Libre de Bruxelles, 1000 Brussels, Belgium; fabrice.journe@umons.ac.be; 4 Department of Human Biology and Toxicology, UMONS Research Institute for Health Sciences and Technology, University of Mons (UMONS), 7000 Mons, Belgium; 5 Department of Human Anatomy and Experimental Oncology, UMONS Research Institute for Health Sciences and Technology, University of Mons (UMONS), 7000 Mons, Belgium

**Keywords:** ginger, foam cells, macrophages, atherosclerosis, oxLDL

## Abstract

Recent findings from the World Heart Federation (WHF) reported a significant increase in cardiovascular disease (CVD)-related deaths, highlighting the urgent need for effective prevention strategies. Atherosclerosis, a key precursor to CVD, involves the accumulation of low-density lipoprotein (LDL) and its oxidation within the endothelium, leading to inflammation and foam cell formation. Ginger extracts, known for their antioxidative and anti-inflammatory properties, show promise in preventing CVD initiation by inhibiting LDL oxidation and reducing foam cell formation. Our results revealed that the active fractions in ginger extracts had antioxidative effects, particularly fractions D and E. Further research is needed to identify the active compounds in these fractions and understand their mechanisms of action. In this context, microfluidic models could offer insights into the effects of ginger on monocyte recruitment in a more physiologically relevant context. Overall, ginger extracts represent a potential novel treatment for preventing CVD initiation, but additional studies are necessary to identify the active molecules in these fractions.

## 1. Introduction

According to a new report from the World Heart Federation (WHF), the number of cardiovascular disease (CVD)-related deaths increased from 12.1 million in 1990 to 20.5 million in 2021 [1]. Despite the growing interest in cardiovascular research over the years, overall mortality and morbidity are still increasing, explaining the importance of pursuing CVD initiation and promoting CVD prevention.

Despite some unknowns, the phenomenon of atherosclerosis is currently well characterized, and the first step involves the accumulation of low-density lipoproteins (LDLs) in the endothelium [2], where they are trapped by biglycans [3] and oxidized by myeloperoxidase and lipoxygenase [4]. These accumulations are preferentially located on bifurcations or curvatures secondary to nonlaminar flow, weakening the endothelium [5]. Through the activation of nuclear factor-kappa B (NF-κB), oxLDL activates endothelial cells, which increases the synthesis of chemokines, adhesion molecules, and differentiating factors, such as macrophage colony stimulating factor (M-CSF) [6].

The binding of endothelial P-selectin and E-selectin to P-selectin glycoprotein ligand-1 (PSGL-1, CD162) on the monocyte surface promotes the rolling of the latter on the endothelium, and the stronger binding of integrins to vascular and intercellular adhesion molecules-1 (VCAM-1 and ICAM-1) on endothelial cells allows monocytes to penetrate through the endothelium [4,5,6,7]. Once they enter the endothelium, monocytes become macrophages, and they are polarized toward the proinflammatory and atherogenic M1 phenotype by Th1 cytokines through the M-CSF pathway [8,9]. M1 macrophages can internalize oxidized LDL (oxLDL) through several scavenger receptors, such as scavenger receptors A and B1 (SR-A and SR-B1), cluster of differentiation 36 (CD36), receptor-related protein low-density lipoprotein 1 (LRP1) or oxidized LDL lectin-like receptor-1 (LOX-1), which turn macrophages into foam cells that then attract more macrophages into the endothelium and inhibit their migration, allowing their retention in plaque formation [9,10,11,12,13]. Finally, vascular smooth muscle cells (VSMCs) can also migrate into plaques and contribute to the formation of necrotic cores [14], which is the cornerstone of major morbidity/mortality, provoking myocardial infarction, stroke, or peripheral ischaemia.

Many of the medications currently used in medicine are derived from plant-based compounds [15]. A large review of natural compounds’ effectiveness on atherosclerosis development demonstrated the impact of many distinct products, notably through their antioxidative activities [16]. Ginger (the rhizome of *Zingiber officinale Roscoe*) has traditionally been used for centuries to treat a series of pathologies, notably stomach pain, asthma, and diabetes [17,18]. Several recent studies have evaluated the antimicrobial and anticancer effects of ginger, as well as its potential as an antiaggregant and hypolipemic agent, the cornerstone of which is its antioxidative activity [19,20]. Most of these pharmacological activities involve non-volatile compounds such as gingerols, zingerone, shogaols, and paradols [18].

Regarding the potential positive effects of ginger on atherosclerosis and CVD, it has been shown that reactive oxygen species (ROS) production could be decreased, as could tumour necrosis factor-a (TNF-a), interleukin-1b (IL-1b), oxLDL oxidation and macrophage absorption [21]. 6-Gingerol can decrease cholesterol synthesis in mice by phosphorylating AMP-activated protein kinase (AMPK), which can inhibit hydroxymethylglutaryl-CoA (HMG-CoA) reductase, as well as vascular smooth muscle cells (VSMCs) synthesis of biglycan, which could hinder LDL fixation on the cell surface [22,23]. Finally, 6-shogaol can activate nuclear factor erythroid 2 (NFE2)-related factor 2 (NRF2), which is known to protect against oxidative stress and decrease LDL oxidation [21].

This information led us to further investigate the potential uses of ginger extracts in the treatment of hyperlipidaemia through a better understanding of these mechanisms.

## 2. Results

### 2.1. Fractionation of Raw Ginger Extract and Thin-Layer Chromatography

**Figure 1:**
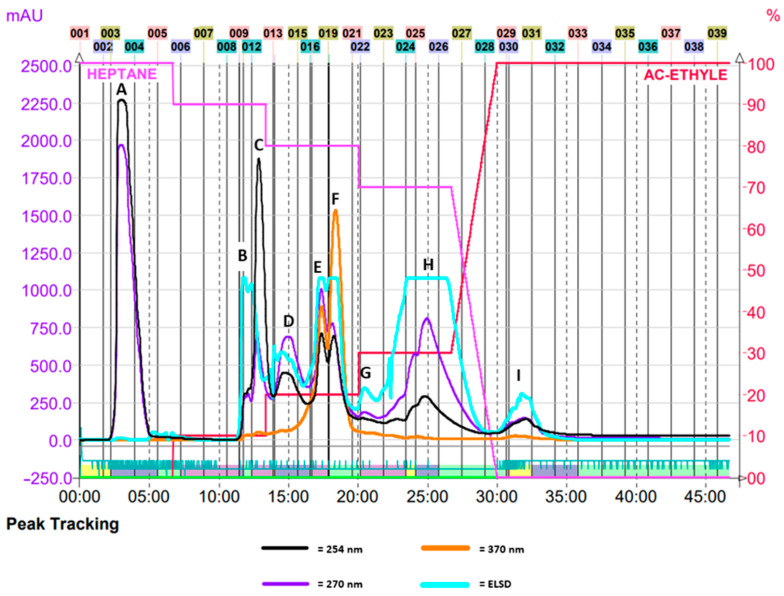
Chromatogram of the fractionation of crude ginger extract by flash chromatography. The horizontal axis represents the elution time (min). The vertical axis on the left corresponds to the absorbance at 270 nm (mAUs = milliabsorbance units), and the axis on the right indicates the solvent gradient (%). Each peak corresponds to a fraction presumably containing different molecules. The evaporative light scattering detector (ELSD) plot shows the nonspecific detection of molecules by light scattering.

The raw extract injected into the flash chromatography system was separated into different fractions—from A to I—according to the detected peaks (Figure 1). Heptane is a nonpolar solvent, and the first fraction is composed of nonpolar components. With the increase in the proportion of the more polar ethyl acetate, the fractions that eluted later were those that mainly contained semipolar molecules.

**Figure 2:**
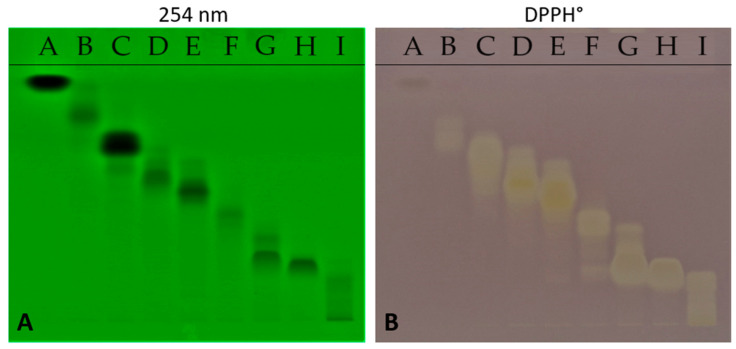
(**A**) HPTLC demonstrating the efficacy of fractionation at 254 nm with nine fractions with different physicochemical properties. (**B**) The same silica gel 60 F254 plate after derivatization with DPPH was used to determine the antioxidative effects of each fraction.

Thin-layer chromatography demonstrated the effectiveness of the fractionation procedure and highlighted the fractions presumably containing larger concentrations of molecules based on the 254 nm absorbance (Figure 2A). The discolouration of the bands after exposure to 2,2-diphenyl 1-picrylhydrazyl (DPPH, Merck Sigma, Darmstadt, Germany) on the plate indicates which fractions present antioxidant potential (Figure 2B). The yellow colour is proportional to the scavenging of the free radical of DPPH and therefore to the antioxidant potential. All the fractions, except fractions A and B, had antioxidant activity at low levels. Fractions D and E, whose bands were strongly discoloured, had the greatest antioxidant potential.

### 2.2. Foam Cell Formation upon oxLDL Exposure

**Figure 3:**
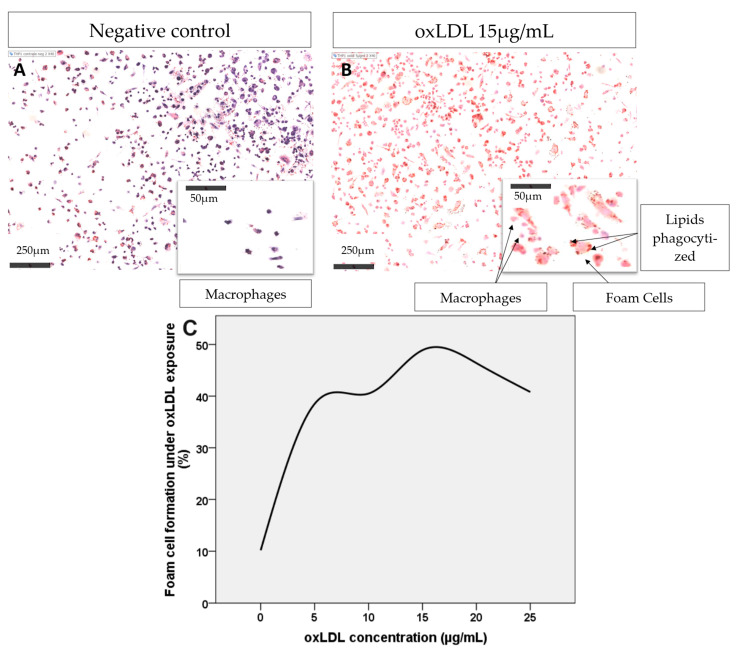
(**A**) THP-1-derived macrophages observed after 48 h of exposure to RPMI medium without oxLDL. (**B**) THP1-derived macrophages observed after 48 h of exposure to RPMI medium containing 15 µg/mL oxLDL. Oil Red O staining. Magnification ×40. Electronic zoom with NDP.scan SQ 1.0 on a NanoZoomer-SQ Digital (Hamamatsu, Japan). (**C**) Line graph representing the evolution of foam cell formation under increasing oxLDL concentration exposition.

To evaluate lipid droplet accumulation, a total of 10.2% of the total THP1-derived macrophages underwent spontaneous foam cell formation after 48 h of incubation in RPMI medium without oxLDL exposure (Figure 3A,B). With increasing oxLDL concentrations of 5, 10, and 15 µg/mL, foam cell formation reached 38.5%, 40.5%, and 48.9%, respectively (Figure 3B). When the concentration was increased to 20 and 25 µg/mL, the foam cell formation decreased to 46.4% and 40.8%, respectively, due to the toxicity of oxLDL to macrophages. Considering these results, we selected a working concentration of 15 µg/mL for the following experiments.

### 2.3. The Effect of Ginger Fractions on Foam Cell Formation

**Figure 4:**
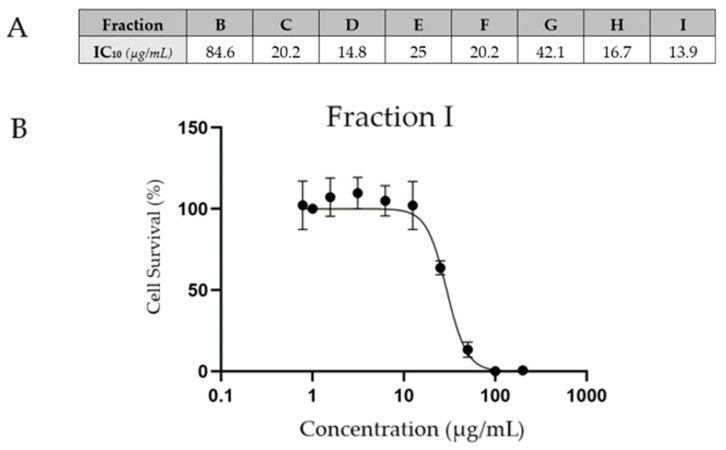
(**A**) The concentration of each fraction that induced 10% cell death after 48 h of exposure was determined via a crystal violet assay. (**B**) Semilogarithmic plot representing the survival of THP-1-derived macrophages upon treatment with increasing concentrations of fraction I.

To limit the toxicity of ginger extracts on macrophages, we assessed the maximal concentration that induces 10% cell death (IC10) to determine the maximal effect of ginger on macrophages through the inhibition of foam cell formation. Given that fraction A has no antioxidative effect, it was no longer considered. The applied concentrations are listed in Figure 4A and were used as the working concentrations for the following assays. The effect of the most active fraction (fraction I) on macrophage survival is presented in Figure 4B.

After 48 h of oxLDL exposure, 48.4 ± 6.0% of the THP-1-derived macrophages were found to be foam cells, concordant with our first observation in Section 2.2.

Fractions B and C were weakly effective, with decreases in foam cell formation to 41.3 ± 9.0% and 36.3 ± 6.8%, respectively (*p* = 0.009 and *p* < 0.001). Fractions E, F, and I were the most effective, with foam cell formation rates decreasing to 24.2 ± 4.8%, 26.4 ± 5.0%, and 27.7 ± 4.9%, respectively (all *p* < 0.001).

**Figure 5:**
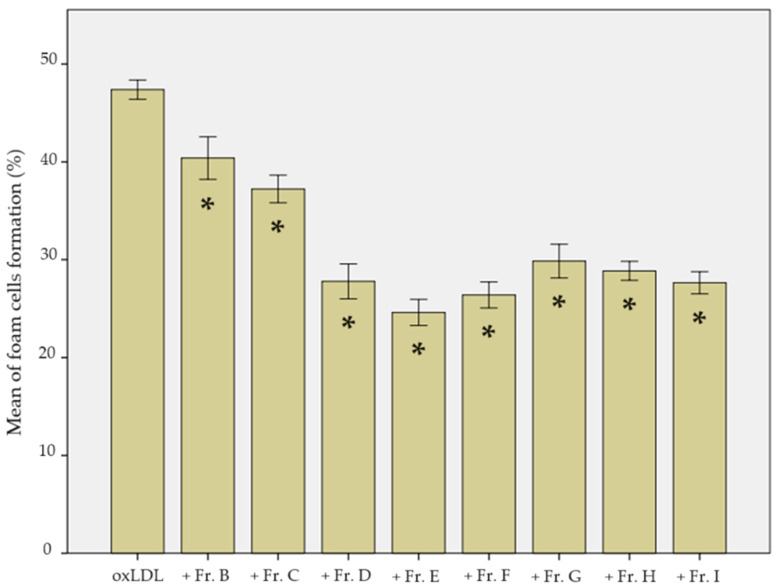
Foam cell formation after 48 h of exposure of THP-1-derived macrophages to oxLDL alone or combined with ginger fractions B to I. Fraction A was not considered after not showing any antioxidant activity (data from 2 biological replicates analysed over 5 microscopic fields each, *n* = 10). Statistical significance was determined with Student’s *t*-test analysis when the *p*-value < 0.05. * shows the statistical difference between the ginger fractions (B to I) and the oxLDL condition.

Finally, fractions D, G, and H induced intermediate effects, with foam cell formation decreasing to 30.2 ± 8.3%, 31.1 ± 7.0%, and 29.4 ± 5.5%, respectively (all *p* < 0.001) (Figure 5).

### 2.4. The Effect of Ginger Fractions on Scavenger Receptors and Cellular Adhesion Markers

The mean fluorescence intensity (MFI) measures the expression of the receptors on THP-1-derived macrophages after various experimental conditions. All conditions were analysed using ANOVA and post hoc Bonferroni’s correction to observe significant differences between the groups.

**Figure 6:**
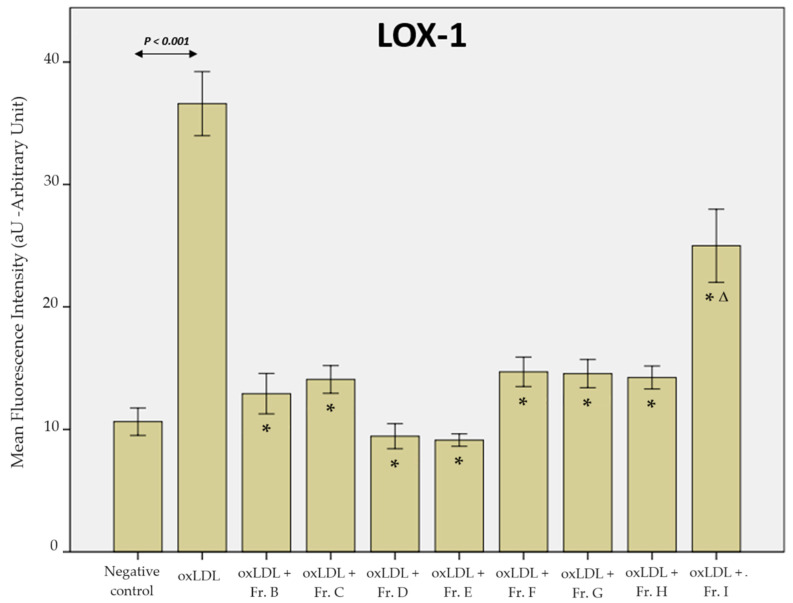
LOX-1 receptor expression: mean fluorescence intensity measured on THP-1-derived macrophages after binding of the LOX-1 antibody. The negative control represents conditions without oxLDL and ginger extract (data from 3 biological replicates analysed over 5 microscopy fields each, *n* = 15). Statistical significance was obtained by ANOVA with Bonferroni’s post hoc correction when the *p*-value < 0.05. * shows the statistical difference between the ginger fractions (B to I) and the oxLDL condition. Δ highlights fraction I, which is statistically different from all other ginger fractions (all, *p* ≤ 0.001).

For the LOX-1 scavenger receptor, the MFI of the negative control condition without oxLDL or ginger extract exposure was 10.6 aU (arbitrary unit). After 48 h of oxLDL exposure, the MFI increased to 36.6 aU (negative control vs. oxLDL, *p* < 0.001). After the addition of ginger fraction B, the MFI decreased to 12.9 aU (Fr. B vs. oxLDL, *p* < 0.001). For fractions C, D, E, F, G, H, and I, the MFIs obtained were 14.1 aU (Fr. C vs. oxLDL, *p* < 0.001), 10.7 aU (Fr. D vs. oxLDL, *p* < 0.001), 9.13 aU (Fr. E vs. oxLDL, *p* < 0.001), 14.7 aU (Fr. F vs. oxLDL, *p* < 0.001), 14.6 aU (Fr. G vs. oxLDL, *p* < 0.001), 14.3 aU (Fr. H vs. oxLDL, *p* < 0.001), and 25.0 aU (Fr. I vs. oxLDL, *p* < 0.001), respectively (Figure 6).

**Figure 7:**
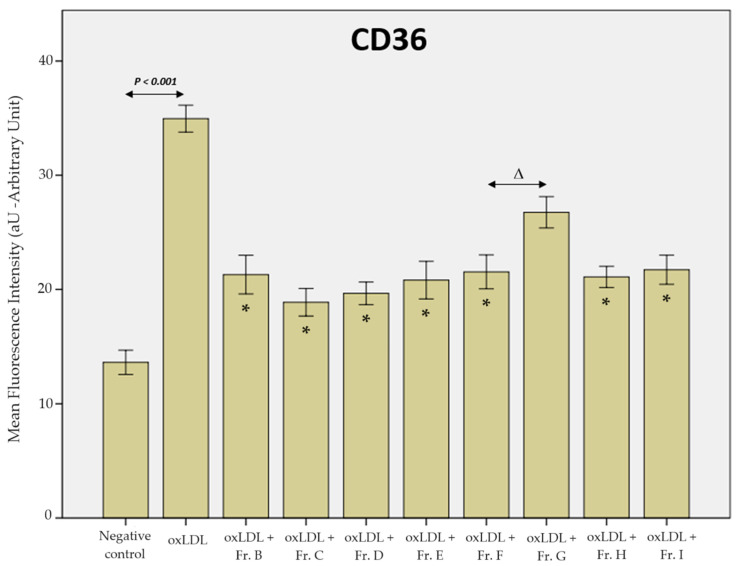
Histogram of the mean fluorescence intensity of the CD36 receptor on THP-1-derived macrophages. The negative control represents conditions without oxLDL or ginger extract. Lack of * represents the only condition (fraction G) that did not reach statistical significance (data from 3 biological replicates analysed over 5 microscopy fields each, *n* = 15). Statistical significance was obtained by ANOVA with Bonferroni’s post hoc correction when the *p*-value < 0.05. * shows the statistical difference between the ginger fractions (B to I, except fraction G) and the oxLDL condition. Δ highlights fraction F, which is the only fraction not reaching statistical difference comparing to fraction G (*p* = 0.069).

The MFI of the negative control condition on the second scavenger receptor, CD36, was 13.6 aU, while the MFI after oxLDL exposure significantly increased to 35.2 aU (negative control vs. oxLDL; *p* < 0.001). All the ginger extracts significantly decreased the MFI of CD36, except fraction G. Thus, fractions B, C, D, E, F, H, and I had MFI values of 20.6 aU (Fr. B vs. oxLDL, *p* < 0.001), 19.8 aU (Fr. C vs. oxLDL, *p* < 0.001), 19.0 aU (Fr. D vs. oxLDL, *p* < 0.001), 20.8 aU (Fr. E vs. oxLDL, *p* < 0.001), 23.3 aU (Fr. F vs. oxLDL, *p* < 0.001), 30.7 aU (Fr. G vs. oxLDL, *p* < 0.001), 22.9 aU (Fr. H vs. oxLDL, *p* < 0.001), and 22.6 aU (Fr. I vs. oxLDL, *p* < 0.001), respectively. Although there was a slight decrease in the MFI, fraction G could not reach a significant difference (*p* > 0.05) (Figure 7).

**Figure 8:**
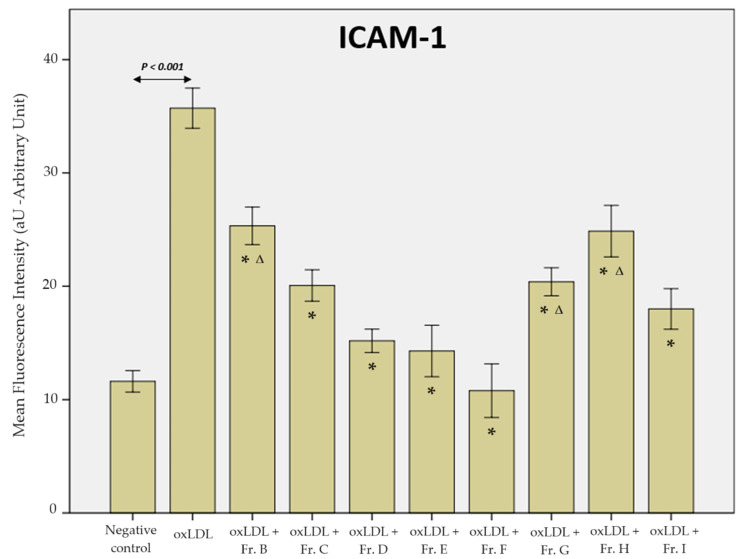
Histogram of the mean fluorescence intensity of the ICAM-1 receptor on THP-1-derived macrophages. The negative control represents conditions without oxLDL or ginger extract (data from 3 biological replicates analysed over 5 microscopy fields each, *n* = 15). Statistical significance was obtained by ANOVA with Bonferroni’s post hoc correction when the *p*-value < 0.05. * shows the statistical difference between the ginger fractions (B to I) and the oxLDL condition. Δ highlights fractions B, G, and H, which are the fractions reaching statistical difference against fraction F, observed as the most effective fraction regarding ICAM-1 expression (all, *p* ≤ 0.001).

oxLDL increased the expression of ICAM-1 with an MFI of 18.8 aU, while the negative control condition had an MFI of 6.4 aU (negative control vs. oxLDL, *p* < 0.001). Once again, fractions B, C, D, E, F, G, H, and I had MFI values of 12.45 aU (Fr. B), 10.0 aU (Fr. C), 7.5 aU (Fr. D), 8.4 aU (Fr. E), 5.4 aU (Fr. F), 12.5 aU (Fr. G), 12.6 aU (Fr. H), and 9.2 aU (Fr. I), respectively, which were significantly increased (*p* ≤ 0.001) (Figure 8).

**Figure 9:**
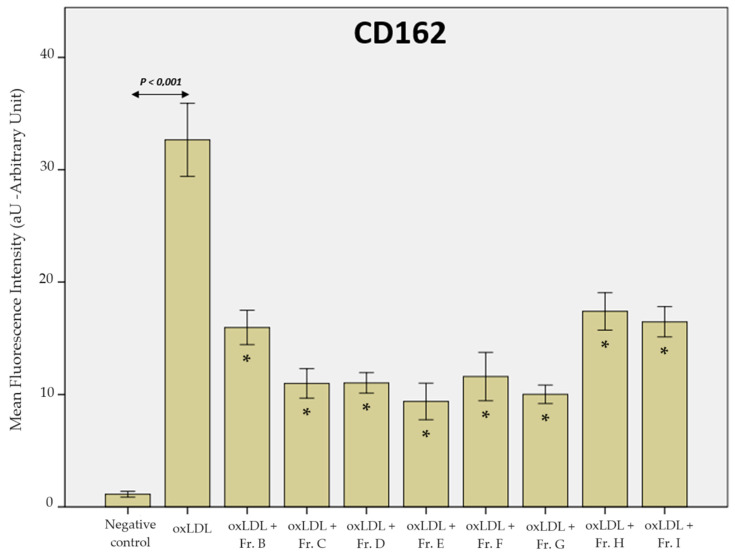
Histogram of the mean fluorescence intensity of the CD162 (PSGL-1) receptor on THP-1-derived macrophages. The negative control represents conditions without oxLDL or ginger extract (data from 3 biological replicates analysed over 5 microscopy fields each, *n* = 15). Statistical significance was obtained by ANOVA with Bonferroni’s post hoc correction when the *p*-value < 0.05. * shows the statistical difference between the ginger fractions (B to I) and the oxLDL condition. No statistical difference against ginger fractions was obtained relative to CD162 expression.

Finally, after 48 h of oxLDL exposure, CD162 expression was increased, as indicated by an MFI of 35.2 aU, compared to that in the negative control group (with an MFI of 13.6 aU; negative control, oxLDL; *p* < 0.001). Like the previous receptors, ginger fractions B, C, D, E, F, G, H, and I had a decreased expression of CD162, as demonstrated by the MFI values of 20.6 aU (Fr. B), 19.8 aU (Fr. C), 19.0 aU (Fr. D), 20.8 aU (Fr. E), 23.3 aU (Fr. F), 30.7 aU (Fr. G), 22.9 aU (Fr. H), and 22.6 aU (Fr. I), all of which were significant (*p* < 0.001) (Figure 9).

### 2.5. Effect of Ginger Fractions on LDL Oxidation

Agarose gel electrophoresis was used to observe the oxidation of LDL. Indeed, as the oxidation rate increases, the electronegativity of these materials increases as well, implying further migration via electrophoresis.

We confirmed that oxLDL migrates further than native LDL and that oxidative modifications are persistent over time, as one-month-old oxLDLs migrate only 2 mm less than freshly produced oxLDLs.

**Figure 10:**
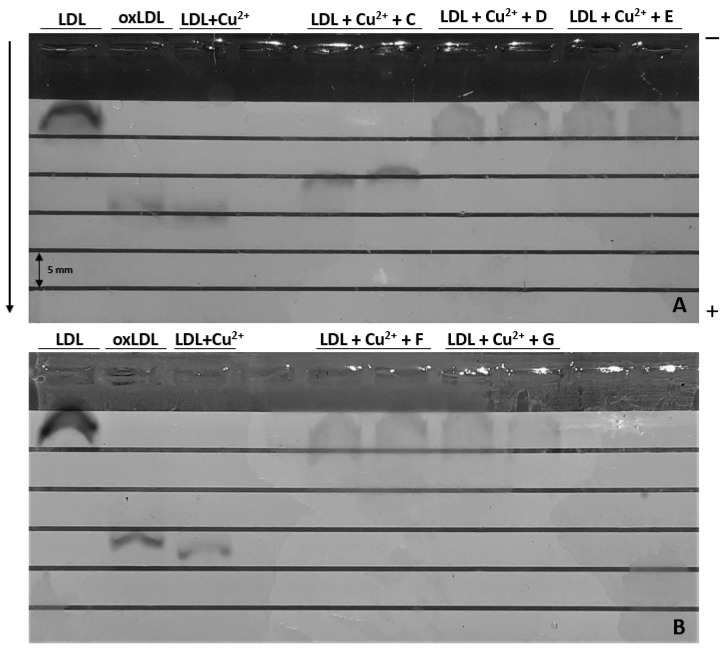
Agarose (0.5%) was used for gel electrophoresis at 70 V for 60 min. Oxidized LDL migrates further than nonoxidized LDL due to increased electronegativity. Freshly oxidized LDL (LDL + Cu²^+^) was compared to one-month-old oxLDL (oxLDL). Freshly prepared LDL was exposed to CuSO_4_ (5 µM) in the presence of ginger extracts C, D, and E (**A**) and to ginger extracts F and G (**B**) (*n* = 2).

When oxLDLs were exposed to ginger fraction C, their migration decreased by 5 mm, while fractions D, E, F, and G migrated exactly as native LDL, showing a better antioxidative effect on LDL (Figure 10). It remains to be determined whether this is a specific LDL protective effect of ginger fractions or a simple scavenging of CuSO_4_-induced ROS. Fractions B, H, and I were not considered due to their lower effectiveness in previous experiments.

### 2.6. The Effect of Ginger Fractions on the Inflammasome

Fractions D and E were selected for their optimal antioxidative effects and their strong positive effects in previous experiments.

**Figure 11:**
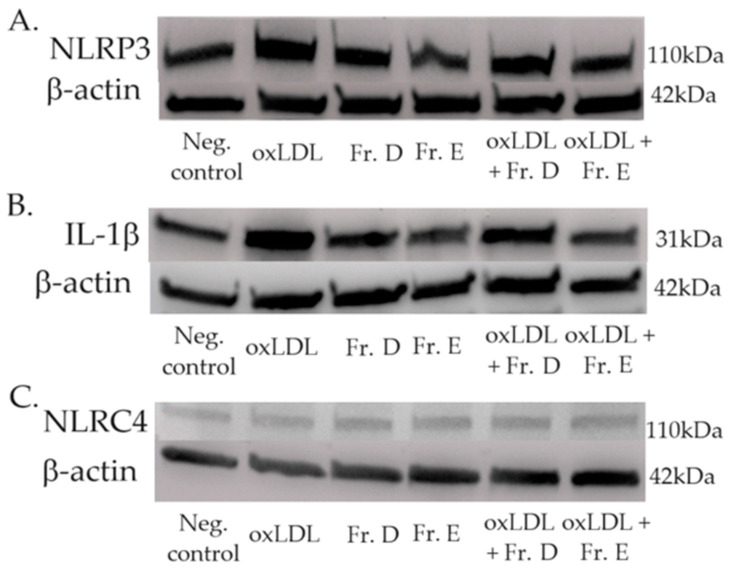
The expression levels of NLRP3 (**A**), IL-1b (**B**) and NLRC4 (**C**) were determined by Western blotting and normalized to their respective β-actin expression levels. The negative control represents untreated THP-1 cells. oxLDL condition represents THP-1 cells treated with 15 µg/mL oxLDL for 48 h, while THP-1 cells were exposed to 14.8 µg/mL of fraction D or 25 µg/mL of fraction E.

The NOD-like receptor family pyrin domain containing 3 (NLRP3) protein is involved in inflammasome signalling via the overexpression of pro-IL-1b [24]. The expression of NLRP3 increased upon oxLDL and LPS exposure (Figure 11A) and induced the overexpression of IL-1b. This was confirmed by our data (Figure 11B).

Both NLRP3 and IL-1b expression were decreased upon exposure to ginger extracts D and E, confirming the antioxidative and protective effects of these fractions.

However, analysis of the NLR family CARD domain-containing protein 4 (NLRC4) did not reveal any significant differences in its expression upon oxLDL and/or ginger exposure (Figure 11C).

## 3. Discussion

Using an in vitro model to assess foam cell formation from THP-1-derived macrophages, these results are in line with previous studies demonstrating the antioxidative effects of ginger, which could result in the prevention of CVD initiation [25,26,27]. Many experiments have reported the effects of specific molecules, such as gingerol or shogaol [22,28], but we decided to fractionate a crude extract to broaden the spectrum of molecules that could have an impact on atherosclerosis while separating, as far as possible, the different compounds in a further bioguided study, to then focus our efforts on the most effective extracts.

Indeed, after thin-layer chromatography (Section 2.1), we discarded fraction A as no antioxidant effect was observed, which is the cornerstone of our observations. Regarding LDL oxidation (Section 2.5), we decided to exclude fractions B, H, and I, as our goal is to concentrate our investigation on the most effective fractions. Finally, when observing the inflammasome expression by Western blotting (Section 2.6), we focused our analysis on fractions D and E, which represent the most effective fractions in all the previous experiments, and with the stronger antioxidative potency. We particularly observed these results in foam cell formation and the LOX-1 and ICAM-1 immunofluorescence.

The scavenging of DPPH on thin-layer chromatoplates indicates a difference in the antioxidant potency according to the fraction, which agrees with subsequent experiments in which lower antioxidant fractions had less capacity to reduce foam cell formation and the expression of pro-atherogenic receptors.

Overall, on the basis of their antioxidant potency, our data indicate that ginger fractions can inhibit the oxidation of LDL, decrease foam cell formation, and prevent an increase in scavenger receptors. All these effects could prevent the formation of new atherosclerotic plaques in human arteries. oxLDL is known to be a cornerstone of atherosclerosis by entering the endothelium and accumulating in macrophages that transform into foam cells and develop the necrotic core of atherosclerotic plaques.

On the basis of the results of the cytotoxicity assay, the majority of the fractions, at the lowest concentrations, increased the cell survival rate compared to that of the control cells. This can be explained by the protective effects of ginger components, which protect cells from basal oxidative stress [29] and prevent apoptosis [30].

We previously described that scavenger receptors are overexpressed on THP-1-derived macrophages in the presence of oxLDL [31], which is in accordance with other findings in the literature [32,33]. The expression of scavenger receptors effectively leads to positive feedback, inducing more foam cells, which produce more chemoattractants, leading to the recruitment of more monocytes to the endothelium. Additionally, the inhibition of PSGL-1 with a blocking anti-P-selectin antibody significantly reduces oxLDL uptake by monocytes [34], which is consistent with the present findings in ginger fractions (Figure 9).

NLRP3 is an intracellular complex that induces the maturation of pro-IL-1b and pro-IL-18 through the activation of Caspase-1, leading to pyroptosis, which results in the release of a large quantity of proinflammatory cytokines that participate in necrotic core formation [35]. By reducing NLRP3 and IL1b expression, ginger fractions decrease the inflammatory status of arteries, probably reducing the formation of necrotic cores [36], but could also have an impact on cardiomyocyte dysfunction, as NLRP3 activates cardiac fibroblasts, which play a role in the progression of heart failure [37]. The potential cardiomyocyte effect should be further evaluated to confirm this hypothesis. Regarding the NLR4 expression under oxLDL and ginger exposition, we sought to observe if the effect would be identical while this inflammasome is activated by bacterial flagellin and bacterial type III secretory system [38]. Those results therefore confirm the selective impact of oxLDL and ginger extract on inflammasome proteins.

It would be pertinent to study whether the mechanism of inhibition of LDL copper oxidation by ginger fractions would be as effective in the presence of myeloperoxidase. Although copper oxidation is the most common way to produce oxLDL in the literature, this is a nonphysiological method that has been shown to cause alterations in both lipids and proteins, while physiological myeloperoxidation mostly induces alterations in proteins [39]. Superoxide anion production could also be monitored, considering its impact on LDL oxidation and atherosclerosis [40]. The overexpression of ICAM-1 by oxLDL leads to increased monocyte migration into the endothelium, increasing monocyte susceptibility to oxidation. As ginger fractions decreased the expression of these proteins, they could disrupt this positive feedback loop and stabilize atherosclerotic plaques.

The precise identification of the active compounds in each ginger fraction is essential for further research and identification of the optimal active molecule that provides the greatest benefit for CVD treatment. Our results tend to highlight fractions D and E as the two most effective fractions of interest, but, interestingly, no statistical differences were highlighted when comparing to some other fractions. The LOX-1 expression after fraction I exposition was significantly different from all other ginger fractions. Regarding CD36 expression, fraction G did not reach statistical significance of reducing expression after oxLDL exposition and was statistically different against all other ginger fractions, except fraction F (*p* = 0.069). No statistical difference against ginger fractions was obtained relative to CD162 expression. Finally, ICAM-1 expression was more heterogeneous as the significance between ginger fractions was complex. Fraction F showed a major reduction in ICAM-1 expression after oxLDL exposure, with a significant difference with fractions B, G, and H.

Further important complementary studies should observe the long-term effects of these compounds to detect potential adverse effects on human cells to transpose them to a potential future long-term anti-atheromatous therapy.

However, research on the mechanisms, other than anti-oxidative, on which ginger compounds influence foam cell formation, endothelial protein expression or inflammasome expression must be deeply explored.

In recent years, interest in the use of microfluidic devices to mimic more physiological conditions has increased in the literature. It would be interesting to repeat our research in a 3D microfluidic model that reconstructs the architecture of a vessel to determine the cause of a decrease in monocyte recruitment.

## 4. Materials and Methods

### 4.1. Fractionation of Raw Ginger Extract and High-Performance Thin-Layer Chromatography

A raw ginger supercritical CO_2_ extract (Zingiber officinale Roscoe, Flavex^®^ Naturextrakte GmbH, Rehlingen-Siersburg, Germany) was fractionated based on its physicochemical properties by flash chromatography (PuriFlash silica hp 15 µm-25G and PuriFlash 415, Interchim^®^, Montluçon, France). Under a pressure of 22 bar, the mobile phase consisted of a gradient of n-heptan and ethyl acetate (Chem-Lab nv, Zedelgem, Belgium) that was stepwise varied every 10 min (0% to 30% by steps of 10%). The different fractions were assembled based on their chromatographic profiles at 254, 270, and 370 nm, yielding a total of 9 fractions.

The first step of solvent evaporation was performed with a rotating evaporator (RotaVapor^®^ R-210, BÜCHI Labortechnik GmbH, Hendrik-Ido-Ambacht, The Netherlands) at 40 °C and 95 mbar to evaporate the ethyl acetate, after which 47 mbar was used to evaporate the n-heptane. The second step of residual solvent evaporation was performed with a centrifugal concentrator (miVac, Genevac™, Ipswich, England) at 40 °C and 18 mbar for 1.5 h.

High-performance thin-layer chromatography (HPTLC) was performed according to the procedure of the *European Pharmacopoeia 11* [41]. Chromatographic layers were HPTLC silica gel 60 F254 plates (Merck Sigma, Darmstadt, Germany). Samples were applied using Automatic TLC Sampler (ATS 4, Camag, Mutenz, Switzerland), tracks with a band length of 8.0 mm, track distance of 11.4 mm, and an application volume of 10 µL. Chromatography was performed in an automatic developing chamber 2 (ADC 2, Camag) with chamber saturation for 20 min under a relative humidity maintained at 33%, development with a mobile phase 70 mm from the lower edge, and drying for 5 min. The mobile phase was n-heptan-diethyl ether (40:60, *v*/*v*). To investigate the antioxidant effect of each ginger fraction, derivatization was performed by applying 3 mL of 0.1% DPPH using a Derivatizer (CAMAG).

### 4.2. oxLDL Production

Home-generated oxLDL was prepared according to our previously described protocol [42]. Human plasma was purchased at the Belgian Red Cross (Croix-Rouge de Belgique, Uccle, Belgium) and investigations conform to the Declaration of Helsinki. Briefly, we isolated LDL and Very-Low-Density Lipoprotein (VLDL) from human plasma by serial precipitation and centrifugation steps. LDL and VLDL were separated by high-performance liquid chromatography, and LDL was oxidized by incubation in 5 µM CuSO_4_ for 20 h. Agarose gel electrophoresis (0.5%) was used to assess the quality of the oxidation.

### 4.3. Cell Culture

The human leukaemia monocytic cell (THP-1, ATCC^®^ TIB202™, Manassas, VA, USA) line was grown in Roswell Park Memorial Institute 1640 medium (RPMI) supplemented with 10% foetal bovine serum (FBS), 5% L-glutamine and 1% penicillin/streptomycin (all provided by Gibco™ Life Technologies, Paisley, UK).

Routine cell culture was carried out in a 5% CO_2_ humidified cell incubator at 37 °C.

### 4.4. Assessment of Foam Cell Formation under oxLDL Exposure by the Oil Red O Assay

THP-1 monocytes were seeded on glass coverslips in a 12-well plate. A total of 150,000 THP-1 cells were incubated with 0.1 µg/mL phorbol myristate acetate (PMA; Merck Sigma, Darmstadt, Germany) for 24 h at 37 °C to induce monocyte-to-macrophage differentiation. After an additional 72 h, the THP-1-derived macrophages were incubated with medium enriched with delipidated and decomplemented charcoal-stripped FBS (Labconsult SA-NV, Brussels, Belgium) containing increasing concentrations of oxLDL, ranging from 0 to 25 µg/mL. Each condition was tested in duplicate.

After 48 h, the cells were fixed with phosphate-buffered saline (PBS) containing 4% paraformaldehyde (PFA; Gibco Life Technologies, Paisley, UK). Nuclei were subsequently stained with hemalum (Klinipath, Olen, Belgium) for 30 s, and the cells were stained with Oil Red O (Merck Sigma, Darmstadt, Germany) to visualize the internalized lipoproteins.

The cells were mounted with Aquatex^®^ (Merck Sigma, Darmstadt, Germany) and observed with an NDP.scan SQ 1.0 on a NanoZoomer-SQ Digital (Hamamatsu, Japan). Foam cell counts were performed using ImageJ 1.53e (Research Services Branch of the National Institute of Health, NIH, Bethesda, MD, USA) on 10 microscopic fields. An optimal oxLDL concentration (15 µg/mL) was selected for the following experiments, i.e., a concentration for which the cytotoxicity is low (~20% of cell death) for a high foam cell count (~50%).

### 4.5. Effect of Ginger Fractions on Foam Cell Formation According to the Oil Red O Assay

The first step was to determine the working concentration for each ginger fraction. THP-1-derived macrophages were seeded on a 96-well plate, as described in Section 4.4, at a concentration of 20,000 cells per well. The medium was replaced with medium supplemented with dilutions of 5 mg/mL ginger fractions dissolved in 5% dimethyl sulfoxide (DMSO) and fresh RPMI medium supplemented with 10% stripped FBS; as ginger extracts are hydrophobic, DMSO was used for dissolution in the medium [43]. The macrophages were exposed to each ginger fraction at concentrations ranging from 0 to 200 µg/mL for 48 h. After fixation with 4% PAF, the cells were stained with a 0.1% crystal violet staining solution (Sigma Aldrich, Saint-Louis, MO, USA). Once the cells were dried, the cell membranes were permeabilized with 0.2% Triton X-100 (Rohm and Haas Company, Philadelphia, PA, USA). The absorbances were measured at 570 nm on a VERSA Max (Molecular Devices, San José, CA, USA). The working concentration for each ginger fraction was selected as the IC10, which means that 10% cell death occurred.

Once the IC10 was determined, 150,000 macrophage-derived THP-1 cells were spread on a glass coverslip in a 12-well dish (polarization step described in Section 4.4). The cells were exposed to the different ginger fractions at the concentrations previously determined. After 4 h of preincubation, 15 µg/mL oxLDL was added for 48 h. Each condition was tested in duplicate.

The cells were fixed with 4% PAF. Nuclei were stained with hemalum, and the cells were stained with Oil Red O. The cells were mounted with Aquatex^®^ and observed with an NDP.scan SQ 1.0 on a NanoZoomer-SQ Digital. Foam cell counts were performed using ImageJ on 10 microscopic fields.

### 4.6. The Effect of Ginger Fractions on Scavenger Receptors and Cellular Adhesion Markers Determined by Indirect Immunofluorescence

THP-1 cells were plated, polarized, and treated with ginger fractions and oxLDL as described in Section 4.4 and Section 4.5. After 48 h of oxLDL and ginger extract treatment, the cells were rinsed with PBS and fixed with 4% PAF. Each condition was tested in duplicate.

For the LOX-1 primary antibody assay (Thermo Fisher Scientific, Waltham, MA, USA), cells were blocked and permeabilized with a solution containing 5% normal goat serum (NGS, R&D Systems, Minneapolis, MN, USA) and 0.3% Triton-X100 in 1× PBS for 1 h at room temperature (RT). The LOX-1 primary antibody was applied to the cells overnight at 4 °C at a dilution of 1/1000 in blocking solution.

For the CD36 primary antibody assay (Merck Sigma, Darmstadt, Germany), the cells were blocked and permeabilized with a solution containing 2% bovine serum albumin (BSA; Sigma Aldrich, Saint-Louis, MO, USA) and 0.3% Triton-X100 in 1× PBS for 1 h at RT. The CD36 primary antibody was applied to the cells overnight at 4 °C at a dilution of 1/100 in blocking solution.

For the ICAM-1 primary antibody assay (Sigma Aldrich, Saint-Louis, MO, USA), the cells were first permeabilized with 0.1% Triton X-100 for 10 min and then blocked in a solution containing 5% NGS in 1X PBS. The ICAM-1 primary antibody was applied to the cells overnight at 4 °C at a dilution of 1/100 in blocking solution.

For the CD162/PSGL-1 primary antibody assay (R&D Systems, Minneapolis, MN, USA), the cells were blocked and permeabilized in a solution containing 5% NGS and 0.3% Triton-X100 in 1X PBS for 1 h at RT. The CD162/PSGL-1 primary antibody was applied to the cells overnight at 4 °C at a dilution of 1/25 in blocking solution.

After several rinses with PBS, the cells were incubated with a secondary antibody for 30 min at RT in the dark and diluted 1/500 in blocking solution. An anti-mouse IgG antibody coupled with Alexa 455 (Invitrogen, Thermo Fisher Scientific, Waltham, MA, USA) was used for CD162/PSGL-1 staining, while an anti-rabbit IgG antibody coupled with Alexa 488 (Thermo Fisher Scientific, Waltham, MA, USA) was used for the other three conditions.

After the final rinses with deionized water, the cells were mounted on glass slides using commercial anti-fading medium (ProLong™ Gold Antifade Reagent with DAPI; Invitrogen, Thermo Fisher Scientific, Waltham, MA, USA). Confocal microscopy was carried out using a Nikon Eclipse Ti2-E inverted microscope (Nikon, Tokyo, Japan). The mean fluorescence intensity (MFI) was determined using ImageJ 1.53e software.

### 4.7. The Effect of Ginger Fractions on the Electronegativity of LDL

To evaluate the ability of ginger extracts to inhibit the oxidation of LDL, we proceeded to agarose gel electrophoresis. As described in our previous protocol, oxLDL migrated further than nonoxidized LDL on an electrophoresis gel due to its increased electronegativity [42].

Briefly, 50 µg/mL LDL was exposed to 5 µM CuSO_4_ (VWR International, Avantor, Radnor, PA, USA) supplemented with ginger extracts C, D, E, F or G. The negative and positive control conditions were native LDL and native LDL + CuSO_4_, respectively. After 20 h, 0.5% agarose gel electrophoresis was performed at 70 V for 60 min.

oxLDLs were stained overnight at RT with Coomassie brilliant blue (SimplyBlue™ SafeStain, Invitrogen™, Carlsbad, CA, USA), after which the gels were rinsed with deionized water for 72–96 h.

### 4.8. The Effect of Ginger Fractions on the Inflammasome by Western Blotting Analysis

THP-1-derived macrophages were plated and exposed to oxLDL and ginger fractions D and E under the same conditions as those described in Section 4.4 and Section 4.5 for 48 h. Only the two most effective ginger extracts were investigated.

After two rinses with Dulbecco’s phosphate-buffered saline (DPBS; Gibco Life Technologies, Paisley, UK), the macrophages were lysed with M-PER Mammalian Protein Extraction Reagent supplemented with protease and phosphatase inhibitors (Halt™ Protease & Phosphatase Inhibitor Cocktail; Thermo Fisher Scientific, Waltham, MA, USA). The lysed cells were centrifuged at 13,000 rpm and 4 °C for 10 min. The supernatants were recovered, and the proteins were quantified versus albumin using a Pierce™ BCA Protein Assay Kit (Thermo Fisher Scientific, Waltham, MA, USA) following the manufacturer’s instructions.

Thirty-five micrograms of proteins were separated on an SDS–polyacrylamide gel (Mini–Protean TGX 4–20%, Bio-Rad Laboratories, Hercules, CA, USA) for 1 h at 120 V. Proteins were transferred to a nitrocellulose membrane using an iBlot^®^ Dry Blotting System (Life Technologies–Invitrogen, Ghent, Belgium). The membrane was stirred for 1 h at RT in a blocking solution containing 5% milk powder (Bio-Rad Laboratories, Hercules, CA, USA) in Tris-buffered saline containing 0.1% Tween (TBS-T; Sigma Aldrich, Saint-Louis, MO, USA).

After three rinses with TBS-T (0.1%), the membranes were incubated overnight at 4 °C on a stirring plate with the NLRC4, NLRP3 or IL-1 primary antibodies (Human Reactive Inflammasome Antibody Sampler Kit II, Cell Signaling Technology, Danvers, MA, USA) diluted 1/1000 in TBS-T (0.1%) and BSA 5%. β-Actin was also detected to allow the standardization of signals. After several rinses, the membranes were incubated in a solution containing secondary antibodies diluted 1/2000 for 1 h at RT on a stirring plate. Then, the Novex™ ECL HRP Chemiluminescent Substrate Reagent Kit (Invitrogen, Thermo Fisher Scientific, Waltham, MA, USA) was used as a reaction solution for 5 min, avoiding light contact. Chemiluminescence was detected with Fusion FX (Vilber, Marne-la-Vallée, France), and band intensities were analysed using ImageJ.

### 4.9. Statistical Analysis

SPSS^®^ Statistics version 23 software (IBM^®^, Armonk, NY, USA) was used for the statistical analysis. Normality was checked using the Shapiro–Wilk test, confirming the applicability of parametric analyses using Student’s *t*-test or ANOVA with Bonferroni’s post hoc correction. The data are expressed as the means ± SDs, with *p* ≤ 0.05 indicating a statistically significant difference.

## 5. Conclusions

Ginger extracts could represent a novel potential treatment for preventing the initiation of cardiovascular disease (CVD) through their antioxidative and anti-inflammatory effects, which are linked to the direct inhibition of the transformation of macrophages into foam cells. Further investigations, including the identification of the active components of fractions D and E, should be conducted to confirm our results.

## Data Availability

The data are contained within the article.
